# 

**Published:** 1902-10-18

**Authors:** 


					the hospital. "The standard of highest purity."?Lancet. Oct. isth, 1902.
CADBURY'S -A M CADBURY'S
cocoa JlPi M <r 1 ooooa
ABSOLUTELY PURS. ^ ? HO DRUQB OR ALKALIBB U8BD.
No. 838. Vol. XXXEII. USS"]
Saturday, Oct. 18th, 1902.
[Sabscription^ms^ pRICH TWOPENCE.

				

